# Stargardt disease: clinical features, molecular genetics, animal models and therapeutic options

**DOI:** 10.1136/bjophthalmol-2016-308823

**Published:** 2016-08-04

**Authors:** Preena Tanna, Rupert W Strauss, Kaoru Fujinami, Michel Michaelides

**Affiliations:** 1 UCL Institute of Ophthalmology, University College London, London, UK; 2 Moorfields Eye Hospital, London, UK; 3 Departments of Ophthalmology, Medical University Graz and Johannes Kepler University, Linz, Austria; 4 National Institute of Sensory Organs, National Hospital Organization, Tokyo Medical Centre, Tokyo, Japan

**Keywords:** Dystrophy, Imaging, Retina

## Abstract

Stargardt disease (STGD1; MIM 248200) is the most prevalent inherited macular dystrophy and is associated with disease-causing sequence variants in the gene *ABCA4*. Significant advances have been made over the last 10 years in our understanding of both the clinical and molecular features of STGD1, and also the underlying pathophysiology, which has culminated in ongoing and planned human clinical trials of novel therapies. The aims of this review are to describe the detailed phenotypic and genotypic characteristics of the disease, conventional and novel imaging findings, current knowledge of animal models and pathogenesis, and the multiple avenues of intervention being explored.

## Introduction

Stargardt disease (STGD1; MIM 248200) is the most common inherited macular dystrophy in both adults and children with a prevalence of 1 in 8000–10 000.^1–7^ STGD1 has an autosomal recessive mode of inheritance associated with disease-causing mutations in the *ABCA4* gene.^8–11^ It is both clinically and genetically highly heterogeneous.^5–7^ ^12–17^

Patients present with bilateral central visual loss, including dyschromatopsia and central scotomata, with characteristic macular atrophy and yellow–white flecks at the level of the retinal pigment epithelium (RPE) at the posterior pole.^1^ ^5^ ^13^ ^18^ ^19^ Onset is most commonly in childhood, with the next peak being early adulthood, and least frequently in later adulthood, with a better prognosis generally associated with a later onset.^2^ ^3^ ^5^ ^7^ ^13^ ^15^ ^16^ ^19^ There is slow progressive loss of retinal function and structure over time; however, there is marked variability both within and between families, suggesting that other important factors influence phenotype, including genetic modifiers and the environment.^12^ ^15^ ^16^ ^20^ ^21^

Although there are currently no proven treatments, there are three main avenues of intervention being explored, with human clinical trials of stem cell therapy, gene replacement therapy and pharmacological approaches.^5^ ^22–25^

### Clinical characteristics

The characteristics of STGD1 vary widely due to the marked phenotypic heterogeneity associated with the large number (>900) of disease-causing sequence variants identified in *ABCA4.*^26^ There are various manifestations of the disease resulting in a spectrum of clinical presentations, rates of progression, imaging, psychophysical and electrophysiological findings, and variable prognosis.

**Figure 1:**
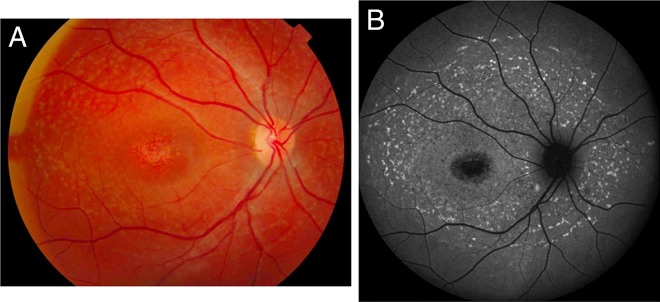
(A) Colour fundus photograph showing typical yellow–white retinal flecks with macular atrophy. (B) Corresponding fundus autofluorescence image showing flecks of both increased and decreased autofluorescence and reduced central macular autofluorescence surrounded by an increased signal.

STGD1 commonly presents as progressive bilateral central vision loss, with onset most often in childhood and a second peak incidence in early adulthood.^3^ ^5^ ^7^ There is increasing evidence that onset relates to the severity of the underlying *ABCA4* variants, with childhood-onset STGD1 being associated with more deleterious variants (including nonsense variants) compared with adult-onset or the later onset ‘foveal-sparing’ (FS) STGD1 (more frequently, missense variants).^3^ ^7^ ^15^ ^16^ ^27^ Initially, ophthalmoscopy can reveal a normal fundus or mild retinal abnormalities (including loss of foveal reflex or mild RPE disturbance) with or without vision loss.^5^ ^16^ The diagnosis can thereby be delayed unless retinal imaging with fundus autofluorescence (FAF) or spectral-domain optical coherence tomography (SD-OCT) and/or electrophysiological assessment (including pattern, full-field and multifocal electroretinography) are undertaken.^3^ SD-OCT will reveal loss of normal architecture that begins at the central macula with relative preservation of the peripheral macula in the first instance and reduced central autofluorescence surrounded by an increased signal or a bull's-eye maculopathy-like appearance on FAF.^13^ ^28^ ^29^ It is important to note that up to a third of children at presentation may not have retinal flecks on fundoscopy or FAF — these develop over time associated with increasing macular atrophy; another reason the diagnosis is often delayed is if retinal flecks are not present (figure 1).^15^ In very early childhood-onset disease with relatively preserved vision, symmetrical yellowish white fine dots at the central macula may also be seen.^30^ ^31^

Although STGD1 is typically diagnosed in childhood or early adulthood, a later age of onset has increasingly been recognised. Late-onset STGD1 (‘FS-STGD1’) is a milder phenotype with a better prognosis and associated with foveal sparing.^15^ ^16^ ^19^ ^32^ Patients with FS-STGD1 often have relatively preserved visual acuity and isolated macular dysfunction (normal full-field electroretinography (ERG)); yet still exhibit the wide phenotypic variability that is characteristic of *ABCA4*-associated retinopathy, including rate of progression—with relative stability in some patients and more rapid progression in others.^15^ ^16^ ^19^ Given that visual acuity may be relatively preserved, static automated perimetry is helpful in detecting and monitoring the pericentral ring-shaped scotoma that may impair reading ability in FS-STGD1.^27^ The mechanism(s) of foveal sparing is unknown; however, given that STGD1 is typically characterised by early foveal degeneration, this phenotype illustrates the potential influence of genetic and environmental factors on the mechanism of cell death; with evidence that specific missense variants may be more frequently observed in FS-STGD1 than non-FS phenotype.^15^ Individual foveal morphological factors such as cone density and macular pigment levels may also play a role in determining the FS phenotype.^33^ ^34^

Electrophysiological assessment including multifocal, pattern and full-field electroretinography can be helpful in confirming the diagnosis of STGD1 and in providing better-informed advice on prognosis. Lois *et al*^17^ established a classification of three phenotypic subtypes of functional loss based on electrophysiological attributes. Group 1 is classified as a severe pattern ERG abnormality (macular dysfunction) with normal full-field ERGs; Group 2 is additional generalised loss of cone function; and Group 3 is additional generalised loss of both cone and rod function.^17^ A longitudinal study, incorporating the cohort of patients used in the aforementioned cross-sectional study to establish ERG group classifications, has now demonstrated that these groups have prognostic implications and that they do not reflect stages of disease; with Group 1 being associated with the best prognosis, Group 3 with the worst and Group 2 with an intermediate variable prognosis.^2^ A total of 22% of patients from Group 1 showed ERG group transition during follow-up, with 11% progressing to Group 2 and 11% to Group 3.^2^ Forty-seven per cent of patients in Group 2 progressed to Group 3.^2^ There was clinically significant ERG deterioration in 54% of all subjects: 22% of Group 1, 65% of Group 2 and 100% of Group 3. Therefore, all patients with initial rod ERG involvement demonstrated clinically significant electrophysiological deterioration; only 20% of patients with normal full-field ERGs showed clinically significant progression.^2^ Such data assist counselling by providing more accurate prognostic information (arguably the most accurate of all inherited retinal diseases) and are also relevant in the design, patient selection and monitoring of potential therapeutic interventions.

### Imaging and disease progression

Imaging techniques providing enhanced assessment of retinal architecture have proven valuable in diagnosis, monitoring and probing the pathogenesis of STGD1, and include FAF, SD-OCT and adaptive optics scanning light ophthalmoscopy (AOSLO).

FAF imaging, by exploiting the autofluorescent properties of lipofuscin and related metabolites, affords assessment of RPE lipofuscin distribution in vivo.^35^ ^36^ FAF has superseded fundus fluorescein angiography (FFA) in the diagnosis of STGD1, which was previously used to identify the ‘dark choroid’ observed in STGD1 due to blockage of underlying choroidal fluorescence by RPE-laden lipofuscin;^7^ though, not all patients have a dark choroid.^18^ Given that STGD1 is characterised by abnormal levels of lipofuscin, FAF imaging may identify fundus changes (early atrophy/flecks/dots) before they are clinically evident on ophthalmoscopy.^3^ ^37^ ^38^ An abnormally reduced FAF signal results from an absence or reduction in RPE lipofuscin density and/or RPE/photoreceptor cell loss.^39^ ^40^ In contrast, an abnormally increased FAF signal derives from excessive lipofuscin accumulation.^40^ Characteristic patterns of FAF, with areas of increased and decreased FAF, are observed in STGD1, and aid both diagnosis and measurement of progression over time.^5^ ^13^ ^41^ Quantified autofluorescence (qAF) has now been developed to indirectly measure RPE lipofuscin in vivo.^42^ Significantly elevated levels of qAF have been detected in STGD1 compared with healthy controls.^42^ A potential concern of qAF and conventional FAF is the use of short-wavelength light which may exacerbate the phototoxicity associated with *ABCA4*-related disease.^43^ In response to this, Cideciyan *et al*^44^ have developed short-wavelength reduced-illuminance autofluorescence imaging that has been also implemented in the largest multicentre longitudinal study about the progression of atrophy secondary to STGD1.^45^

Several studies have retrospectively evaluated longitudinal FAF changes and patterns in STGD1. The largest published series to date (n=68) classified patients into three FAF subtypes at baseline: type 1 had a localised low signal at the fovea surrounded by a homogeneous background (n=19), type 2 had a localised low signal at the macula surrounded by a heterogeneous background with numerous foci of abnormal signal (n=41) and type 3 had multiple low-signal areas at the posterior pole with a heterogeneous background (n=8).^13^ The areas of reduced AF signal were measured, and the rate of atrophy enlargement (RAE) was calculated, with the mean follow-up interval being 9.1 years. The RAE (mm^2^/year) based upon baseline AF subtypes was significantly different: 0.06 in type 1, 0.67 in type 2 and 4.37 in type 3. There was a significant association between AF subtype and genotype, with a preponderance of milder sequence variants (including missense) in type 1 and more deleterious sequence variants (including nonsense) being identified in type 3.^13^ The observation that AF pattern at baseline influences the enlargement of atrophy over time and has genetic correlates assists in the provision of counselling on prognosis in STGD1 and is valuable for clinical trials.

**Figure 2:**
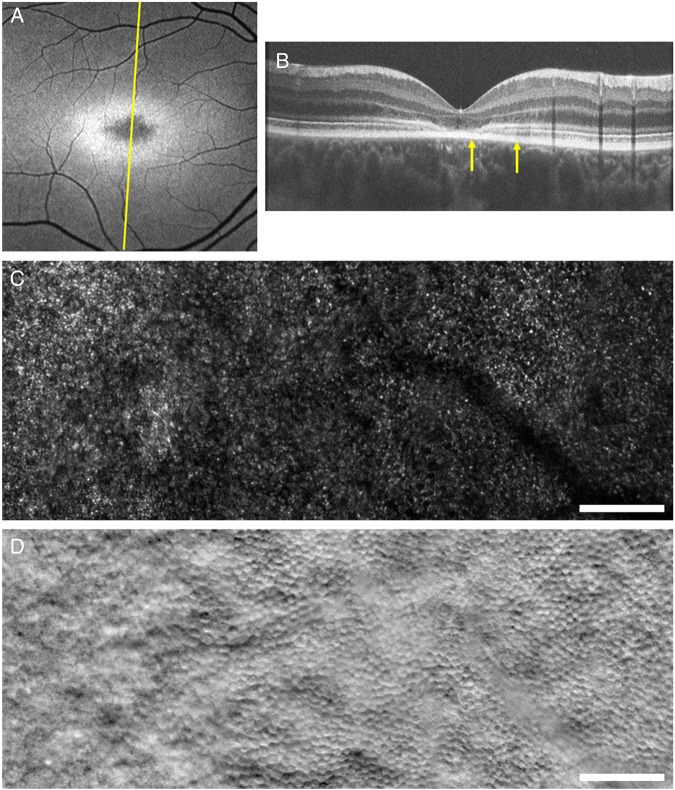
Multimodal imaging of the right eye of a molecularly proven 15-year-old patient with Stargardt disease. (A) Fundus autofluorescence image. The yellow line indicates the scanning level of the optical coherence tomography (OCT) scan in (B). (B) OCT scan showing central loss of outer retinal structure. The yellow arrows indicate the location and extent of the adaptive optics scanning light ophthalmoscopy (AOSLO) montages in (C) and (D) through the transition zone. (C) Confocal AOSLO montage of the photoreceptor mosaic. (D) Split-detection AOSLO montage of the photoreceptor mosaic. The far left side of (C) and (D) is closer to the fovea and lacks cone structure, corresponding with the lack of outer hyper-reflective layers in (B). Moving towards the right of (C) and (D), away from the fovea and superiorly on the retina, (C) shows structural changes that are relatively challenging to interpret, whereas (D) clearly shows the presence of cones. Scale bars represent 100 µm.^48^

SD-OCT provides high-resolution cross-sectional images of retinal lamination and allows early detection of foveal outer retinal loss, which is the hallmark of childhood- and adulthood-onset STGD1 (figure 2). The earliest OCT abnormality which has been detected in children, as young as 5 years of age, is external limiting membrane thickening, prior to the development of atrophy.^30^ ^31^ ^46^ ^47^ Serial OCT imaging may be employed to monitor progression by measuring changes in several parameters including total retinal thickness and macular volume, outer retinal thickness, outer nuclear layer (ONL) thickness and inner segment ellipsoid loss.^28^ For the purposes of clinical trial end-point validation, the demonstration of structure–function correlation will also likely be necessary.^5^ It is noteworthy that it has been shown that central foveal thickness on OCT correlates with visual acuity loss.^27^

AOSLO allows in vivo cellular imaging, with custom-built research systems now able to visualise rods, cones—including foveal cones––and RPE mosaics (figure 2).^48^ Both confocal and split-detector (SD; non-confocal) AOSLO imaging can be undertaken.^48^ Confocal imaging requires relatively intact photoreceptor outer segment structure to detect wave-guiding photoreceptor cells. This thereby results in a significant number of ‘dark spaces’ observed in the cone mosaics of patients with STGD1, where it was unknown until the advent of SD-AOSLO whether these dark spaces lacked an optical signal due to lack of cones or whether cones were present but not wave-guiding.^33^ ^47^ ^49^ ^50^ This has fundamental implications for determining participant suitability and selection for intervention, and thereby clinical trial design and the likelihood of detecting safety and efficacy signals robustly and sensitively. In direct contrast, SD imaging does not require intact outer segments, but affords imaging of *inner* segments. The dark spaces have now been shown in many cases to harbour inner segments, although often ‘swollen’ compared with normal cone inner segments, suggesting there are more cones than previously imaged with confocal techniques that could potentially be rescued with novel therapies, including gene replacement. Moreover, SD imaging has identified cones that would not have been predicted on the basis of OCT alone, suggesting that AO imaging may be a more sensitive measure of photoreceptor integrity.^48–50^ However, these pilot observations need to be applied to large cohorts of molecularly proven patients over time, including quantitative AOSLO-derived measurements to determine disease progression on a cellular level over time, including foveal and parafoveal (‘transition zone’) cone densities and cone spacing.^33^ ^47^ ^49^ Structure–function correlations will also be valuable—with abnormal cone spacing having been previously shown to correlate to abnormal FAF and reduced visual function.^47^

The vast majority of data to date on disease progression is retrospective from single centres and thereby inherently limited. There is a need for prospective natural history studies of large cohorts of molecularly proven patients. One such ongoing multicentre international study is the ‘Progression of Atrophy Secondary to Stargardt Disease (ProgStar)’ study that characterises disease progression using psychophysical measures (such as mesopic and scotopic microperimetry) and imaging (SD-OCT and FAF) to establish endpoints for clinical trials of emerging therapies.^5^

### Molecular genetics

STGD1 is inherited in an autosomal recessive fashion and is caused by sequence variants in the gene *ABCA4,* with the carrier frequency believed to be up to 1 in 20.^51^ Disease-causing variants in *ABCA4* are also associated with cone dystrophy, cone–rod and ‘rod–cone’ dystrophy. *ABCA4* is a large, highly polymorphic gene, consisting of 50 exons, with over 900 disease-associated variants reported to date.^9^ ^10^ ^26^ ^52–54^ This highly polymorphic nature and large number of variants make ascribing definite disease-causation problematic; moreover, the vast allelic heterogeneity makes genotype–phenotype correlations very challenging indeed. In general, missense variants are associated with milder, later onset disease, while null alleles are associated with more severe, earlier onset disease; however, certain missense variants can also have severe functional effects similar to nulls (eg, p.Leu541Pro and p.Ala1038Val (complex), and p.Arg1640Trp).^14^ ^55^ ^56^ The interaction between variants may also affect the functional outcome; while there is evidence that p.Gly1961Glu in the homozygous state typically causes a milder phenotype, a more severe phenotype results when associated with various additional *ABCA4* mutations.^14^ ^57^ Certain missense variants appear to be more commonly observed in the mildest *ABCA4*-associated phenotype, FS-STGD1, including p.Arg2030Gln.^15^ ^58^

*ABCA4* is a member of the ABC transporter gene superfamily, encoding the retinal specific transmembrane ABCA4 protein, a member of the ATP-binding cassette transporter superfamily.^59^ ^60^ ABCA4 is localised to the rim of the rod and cone outer segment discs, and is involved in the active transport of retinoids from photoreceptor to RPE.^59–61^

The high allelic heterogeneity makes molecular genetic testing of *ABCA4*-associated retinal disease very challenging. It has been reported that direct Sanger sequencing of the entire *ABCA4* coding region detects between 66% and 80% of disease-causing alleles; however, this is not practically or economically possible in large cohorts.^58^ ^62^ Arrayed primer extension (APEX) technology, which screens for all known previously reported *ABCA4* variants, detects approximately 65% to 75% of all disease-associated alleles.^63^ ^64^ However, by definition, novel variants are not detected by APEX technology, necessitating the use of other methodologies for high-throughput systematic screening of the entire coding region, especially in cases where one or both disease-causing alleles have failed to be identified by the array. A high-throughput strategy based on next-generation sequencing has now been shown to be highly effective in detecting both alleles, including new *ABCA4* variants not included in the APEX array.^9^ ^10^ The identification of both disease-causing alleles improves the accuracy of diagnosis and the counselling of patients, and also assists in more effective patient selection of genetically confirmed participants for current and future clinical trials.

### Pathogenesis and animal models

The visual cycle consists of enzyme-catalysed reactions converting all-*trans* retinal, derived from the photobleaching of rhodopsin and cone opsin, back to 11-*cis* retinal.^60^ ^65^ All-*trans* retinal is released from the light-activated rhodopsin or cone opsin into the rod and cone outer segments, respectively, and forms a complex with phosphatidylethanolamine (PE) resulting in N-retinylidene-phosphatidylethanolamine (N-ret-PE), which is transported to the disc surface by ABCA4. The lack of, or inefficient removal of, N-ret-PE from photoreceptor outer segments, secondary to ABCA4 dysfunction/mislocalisation, results in an accumulation of bisretinoid compounds in outer segment discs, and ultimately in toxic levels of bisretinoid A2PE in photoreceptor membranes.^43^ ^60^ ^65^ A2PE is hydrolysed to form the highly toxic metabolite A2E, which accumulates as a component of lipofuscin in RPE cells, and ultimately results in RPE dysfunction and death, with subsequent photoreceptor dysfunction/loss.^43^ ^66^ ^67^ While the commonly accepted RPE failure followed by subsequent photoreceptor cell dysfunction/death may be the sequence of events in the majority of *ABCA4*-asociated disease, it may not be the case in, for example, FS-STGD1.^15^

While the STGD1 mouse model (*ABCA4* knockout) has significant limitations, including that mice lack a macula (the primary area affected in STGD1) and the disease in mice is of later onset and exhibits slower degeneration than that seen in patients, it has helped shed light on the aforementioned underlying pathogenesis. Significantly elevated retinal levels of A2E and other lipofuscin fluorophores have been identified in this model.^61^ The *ABCA4* knockout mice show delayed dark adaptation, increased all-*trans*-retinaldehyde following light exposure, increased PE in outer segments and accumulation of N-ret-PE. Moreover, they have an accelerated deposition of lipofuscin and A2E in the RPE, supporting ABCA4's role as a transporter of N-ret-PE across disc membranes.^68^ The finding that the retinal degeneration in the knockout mouse was accelerated with exposure to significantly bright (ultraviolet) light^69^ ^70^ and ingestion of large doses of vitamin A^43^ ^71^ has led to patients with STGD1 being advised to avoid excessive exposure to bright sunlight and wear good-quality sunglasses with UVA/UVB blocking properties, and to also avoid vitamin A supplementation. Interestingly, in a light exposure study, five patients with STGD1 who wore a black contact lens in one eye during waking hours for 12 months were observed to have less progression on FAF imaging in their study eye compared with the fellow eye.^72^

### Avenues of intervention

STGD1 is currently subject to more clinical trials than any other inherited retinal disease, with gene replacement, stem cell therapy and pharmacological approaches.

Gene replacement therapy targets viable photoreceptors, with the underlying aim being to slow or prevent further retinal degeneration; so, the earlier the intervention the better. Adeno-associated virus (AAV) vectors have been the gene transfer system of choice for human gene therapy, including retinal diseases.^73^ ^74^ However, a major limitation is that *ABCA4* is larger than the current AAV vector capacity, a challenge that needs to be addressed for other genes that commonly cause inherited retinal disease, including *USH2A.*^75^ Subretinal injection of a lentivirus vector delivering *ABCA4* has therefore been developed, given the larger cargo capacity of lentiviruses, and is currently in an ongoing Phase I/II clinical trial (ClinicalTrials.gov Identifier: NCT01367444). There have been no safety concerns in the first three cohorts of subjects with relatively advanced disease, and no definite evidence of efficacy, with the final cohort with less severe disease now being recruited, with arguably more potential to show benefit.^22^ ^24^ ^76^

In view of the fact that RPE cell dysfunction/loss is believed to precede photoreceptor cell dysfunction/loss in STGD1 and that RPE cells can be generated in the laboratory relatively easily, a Phase I/II stem cell therapy trial (ClinicalTrials.gov Identifier: NCT01469832) has been undertaken using human embryonic stem cell derived RPE cells transplanted subretinally in patients with severe advanced STGD1.^23^ There have been no safety concerns to date, and efficacy data are awaited. It remains possible that RPE cell replacement alone will be insufficient and that photoreceptor cells will need to also be transplanted.

There are many drugs that are already available or have been specifically developed that target different aspects of the visual cycle (vitamin A recycling pathway) and may thereby be potentially beneficial in slowing or stopping progression in STGD1.^65^ These include agents aimed at either (i) reducing the formation of toxic by-products of the visual cycle, by reducing delivery of vitamin A or inhibition of various enzymes participating in the visual cycle, or (ii) directly targeting toxic metabolites such as A2E. One of these drugs, which aims to allow normal visual cycle function and yet stop N-ret-PE and A2E formation, is a chemically modified vitamin A taken orally that will compete with dietary vitamin A, normally enter the visual cycle, but will not dimerise and therefore not exit the visual cycle to generate A2E.^25^ ^77^ ^78^ This is currently being investigated in a Phase II clinical trial (ClinicalTrials.gov Identifier NCT02402660).

## Conclusions

STGD1 is one of the most common causes of inherited childhood and adulthood visual impairment, with inherited retinal disease now being the most common cause of certifiable blindness in the working age population in England and Wales and the second most common in childhood.^79^ STGD1 is both phenotypically and genetically highly heterogeneous with significant advances having been made in our ability to identify the disease at the earliest stages, characterise clinical features that allow better-informed advice on prognosis, perform accurate rapid molecular genetic testing, and in our understanding of underlying disease mechanisms. These developments have allowed multiple clinical trials to be currently ongoing with many more, especially drug trials, anticipated over the next 5–10 years. Further robust longitudinal prospective natural history studies, probing genotype–phenotype and structure–function associations, are crucial in order to provide improved prognostication and genetic counselling, as well as optimisation of clinical trial design, including identifying suitable participants, windows of opportunity and the most sensitive and reliable outcome metrics.
